# Silent Bird Poisoning in Poland: Reconfirmation of Bromadiolone and Warfarin as the Proximal Causes Using GC-MS/MS-Based Methodology for Forensic Investigations

**DOI:** 10.3390/ph17060764

**Published:** 2024-06-11

**Authors:** Damian Kobylarz, Łukasz Paprotny, Dorota Wianowska, Maciej Gnatowski, Kamil Jurowski

**Affiliations:** 1 Department of Regulatory and Forensic Toxicology, Institute of Medical Expertises, ul. Aleksandrowska 67/93, 91-205 Łódź, Poland; 2 Center Shim-pol Company, ul. Lubomirskiego 5, 05-080 Izabelin, Poland; 3 Department of Chromatography, Institute of Chemical Sciences, Faculty of Chemistry, Maria Curie-Skłodowska University in Lublin, Pl. Maria Curie-Skłodowska 3, 20-031 Lublin, Poland; 4 Research and Development Centre, ALAB Laboratories, ul. Ceramiczna 1, 20-150 Lublin, Poland; 5 Laboratory of Innovative Toxicological Research and Analyzes, Institute of Medical Studies, Medical College, Rzeszów University, Al. mjr. W. Kopisto 2a, 35-959 Rzeszów, Poland

**Keywords:** rodenticides, GC-MS/MS, anticoagulant coumarin derivatives, warfarin, bromadiolone

## Abstract

The extensive use of rodenticides poses a severe threat to non-target species, particularly birds of prey and scavengers. In this study, a GC-MS/MS-based method was used to unlock the cause of bird deaths in Poland. Organs (liver, heart, kidney, and lungs) collected during autopsies of two rooks (*Corvus frugilegus*) and one carrion crow (*Corvus corone corone*), as well as fecal samples, were analyzed for the presence of anticoagulant coumarin derivatives, i.e., warfarin and bromadiolone. As for warfarin, the highest concentration was found in crow samples overall, with concentrations in the feces and lungs at 5.812 ± 0.368 µg/g and 4.840 ± 0.256 µg/g, respectively. The heart showed the lowest concentration of this compound (0.128 ± 0.01 µg/g). In the case of bromadiolone, the highest concentration was recorded in the liver of a rook (16.659 ± 1.499 µg/g) and this concentration significantly exceeded the levels in the other samples. By revealing the reality of the threat, these discoveries emphasize the need to regulate and monitor the trade in rodenticides.

## 1. Introduction

Anticoagulant rodenticides are compounds commonly used around the world to reduce rodent populations. Their mechanism of action is through blocking the activation of vitamin K, causing coagulation disorders and hemorrhages leading to death [1]. Warfarin is a representative of the first generation of anticoagulants. To produce the expected effect (rodent death), its repeated consumption is necessary as a result of the characteristic delayed non-lethal effect. In turn, bromadiolone is a second-generation anticoagulant rodenticide. Compared to warfarin, it is more effective due to the prolonged period of toxic effects resulting from its ability to accumulate in the body, especially in the liver [2]. Unfortunately, this increased effectiveness has the side effect of accidentally poisoning non-target animals [3].

The literature contains several reports of indirect poisoning with rodenticides in various predatory animals, both wild and domestic, due to the use of these compounds in animals constituting the beginning of their food chain [2,4,5]. For example, in 2022, an analysis of 40 poisonings of the white-tailed eagles (*Haliaeetus albicilla*), which is under strict protection in Poland due to the risk of extinction, was described by Sell et al. [5]. The analyzed cases concerned the years 2018–2020. In this study, liver samples from birds suspected of rodenticide poisoning were analyzed using liquid chromatography coupled to mass spectrometry. In all examined cases, at least one of the rodenticides, bromadiolone, brodifacoum, difenacoum, or flocoumafen, was detected, and the average concentration of their sum was 174.4 µg/kg (from 2.5 to 1225.0 µg/kg). Moreover, it was found that in 10% of cases the direct cause of death was rodenticide poisoning. The concentration range detailed in the study corresponds to lethal or toxic concentrations. As researchers point out, determining the cause of poisoning in birds of prey is often challenging, particularly when trying to establish anticoagulant rodenticides (AR) as the direct cause of death. There is no universally accepted threshold to conclusively diagnose AR poisoning due to the significant variability in sensitivity among species, races, and even individual birds. However, some researchers propose species-specific thresholds, with suggested lethal levels often cited as >100 μg/kg or >200 μg/kg for birds of prey. In this research on white-tailed eagles, researchers observed blood clotting disorders frequently when the combined AR concentration exceeded 100 μg/kg [6,7].

From an ecological point of view, birds are an important element of food webs, due to their role in regulating the populations of various animals. Therefore, the widespread use of rodenticides, especially coumarin derivatives, raises serious environmental concerns and makes birds a key object of research on the toxicological effects of these compounds [5,8,9,10,11]. However, in addition to poisonings of predatory animals, clinical cases of poisonings in humans are also described in the literature [12]. This type of poisoning most often occurs after oral ingestion, although poisoning through the skin and mucous membranes also occurs. Typical symptoms of poisoning in humans are bleeding from the mucous membranes, urinary and gastrointestinal tracts, but atypical symptoms may also occur, such as central nervous system symptoms due to intracranial hematomas, which can also have fatal consequences. 

With the above in mind, our research, intended for the justice system and law enforcement agencies, focuses on the toxicological analysis of bromadiolone and warfarin in the organs of birds (liver, heart, kidney, and lungs) as well as in fecal samples after acute poisoning. Their aim is to uncover the “Silent Bird Poisoner in Poland”—the frequently unnoticed and sometimes intentional act of poisoning animals. To achieve this, we utilize the “gold standard” analytical method, GC-MS/MS, particularly valuable for forensic investigations due to its extensive libraries of mass spectra.

## 2. Results

**Figure 1:**
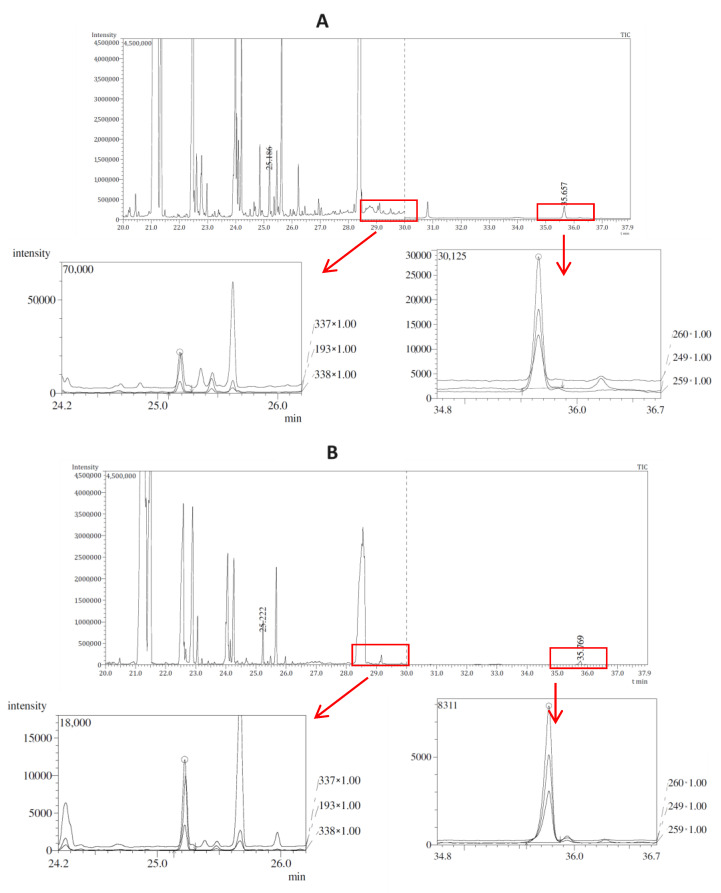
Gas chromatograms obtained during the analysis of a real stool sample (sample no. 1, (**A**)) and a heart sample (sample no. 2, (**B**)) with enlarged fragments of SIM chromatograms and the peaks of warfarin (retention time 25.2 min) and bromadiolone (retention time 25.2 min) marked on them.

Exemplary chromatograms obtained in optimized conditions for authentic test samples, on the example of the analysis of a stool sample (sample no. 1) and a heart sample (no. 2), are presented in Figure 1 in parts A and B, respectively. In each part, below the TIC chromatogram with marked integration windows, there is an enlarged SIM chromatogram for warfarin and bromadiolone, respectively. These compounds were detected based on the presence of fragment ions of 337, 193, 338 *m*/*z* for warfarin and 260, 249, 259 *m*/*z* for bromadiolone, respectively, and were confirmed through the consistency of the signal intensity ratios of the given ions in the chromatograms obtained for the tested samples and the standard solution and the compliance of the obtained retention indices compounds with data contained in the NIST14 library for a given substance. The analysis of the presented data confirms that the GC-MS/MS conditions used allow for both qualitative and quantitative analysis of warfarin and bromadiolone in natural samples.

**Figure 2:**
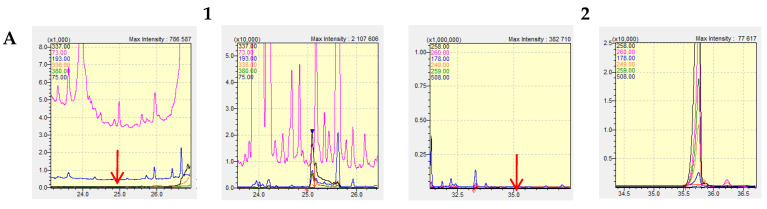

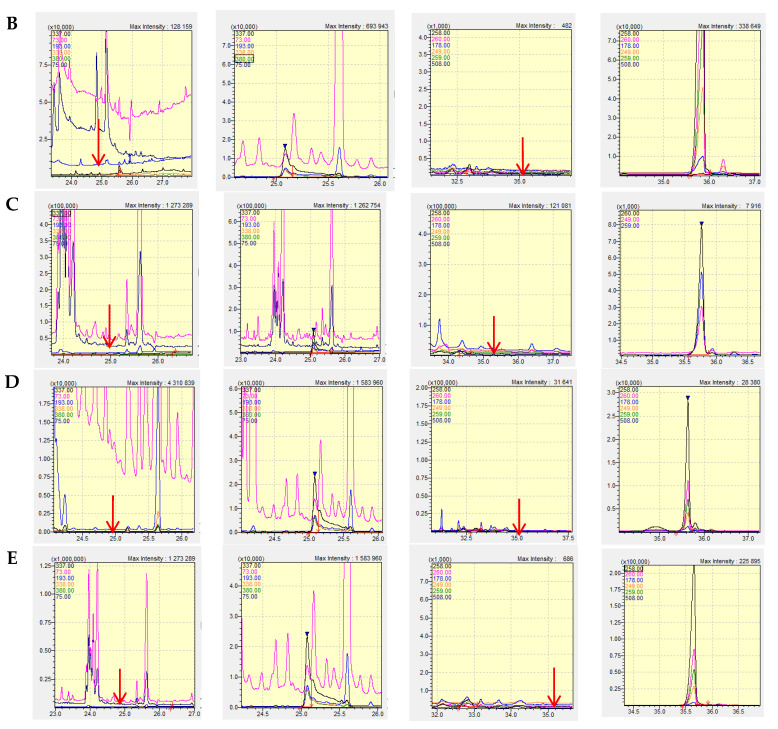
SIM chromatograms obtained during validation experiments for five tested blank matrices (**A**–**E**), each of which was spiked with warfarin (**1**) and bromadiolone (**2**) at a level of 5 ppm. In the chromatograms of blank matrices, the retention time of the appropriate analyte (in its absence) is marked with an arrow.

In order to estimate the analytical utility of the described method, its validation procedure was performed according to the general validation criteria [13]. Examples of chromatograms obtained in this series of tests are shown in Figure 2. The results of the validation experiments are gathered in Table 1. The presented data clearly show that the developed method is characterized by good linearity, very low detection limits and satisfactory inter- and intra-day precisions for warfarin and bromadiolone analysis in natural biological samples.

**Figure 3:**
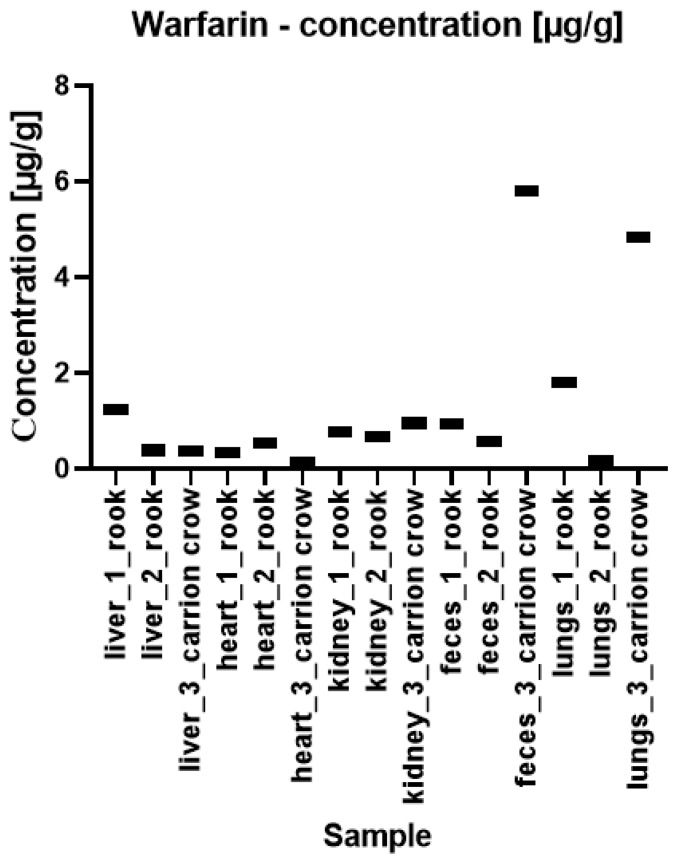
Concentrations of warfarin in forensic samples of rooks (marked as 1 and 2) and carrion crow (3).

**Figure 4:**
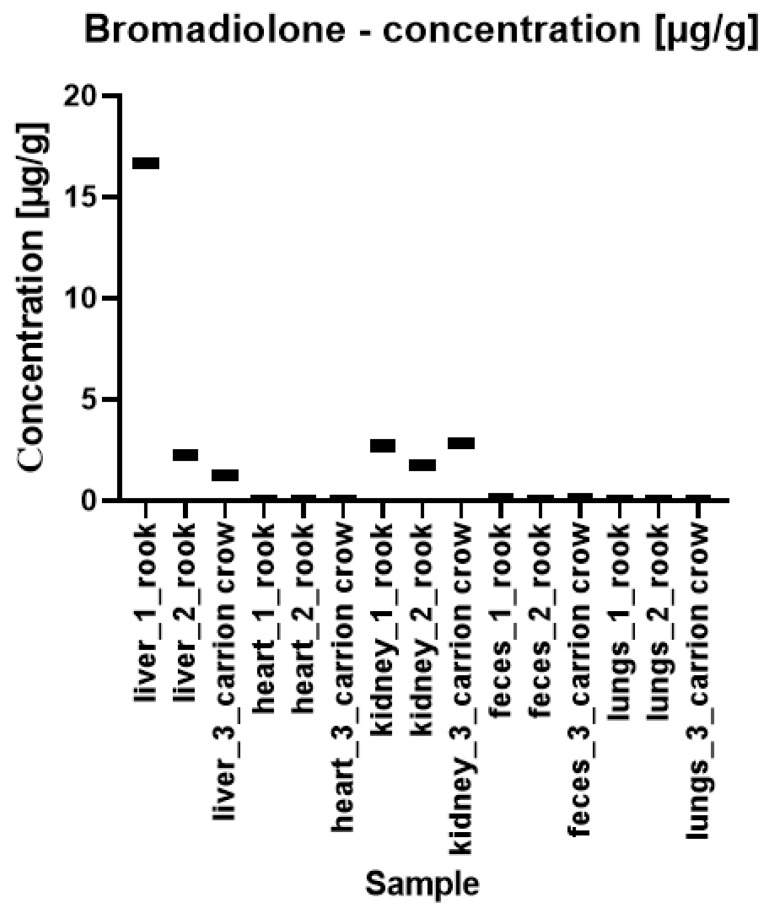
Concentrations of bromadiolone in forensic samples of rooks (marked as 1 and 2) and carrion crow (3).

The quantitative results of the content of warfarin and bromadiolone in bird tissue and feces samples are presented in Figure 3 and Figure 4, respectively. As you can see, bromadiolone and warfarin were detected in each of the analyzed samples. 

The highest concentration of warfarin was detected in the carrion crow’s feces at 5.812 ± 0.368 µg/g (sample no. 3 in Figure 3). Slightly lower concentrations were found in the lung sample—4.840 ± 0.256 µg/g. Interestingly, the carrion crow heart had the lowest warfarin concentration of all samples tested, 0.128 ± 0.01 µg/g. Regarding bromadiolone (see Figure 4), the highest concentration was detected in a rook’s liver at 16.659 ± 1.499 µg/g, which is a particularly significant level compared to the concentrations of 2.241 ± 0.193 µg/g and 1.229 ± 0.019 µg/g found in the other two samples. The fecal samples from the three birds showed the lowest concentrations of 0.001 ± 0.001 µg/g, 0.001 ± 0.001 µg/g and 0.003 ± 0.001 µg/g, and the heart of the carrion crow showed a concentration of 0.004 ± 0.001 µg/g. Overall, statistically significant differences in compound concentrations were observed between subjects. Despite this variability, some patterns emerged, particularly for bromadiolone. This substance showed the highest concentrations in the liver and kidneys, while its concentration in the heart and lungs was significantly lower. Unlike bromadiolone, warfarin did not show such clear discrepancies between tissues. However, the differences between the individuals were apparent. In the carrion crow, the highest concentrations of warfarin were found in the lungs, feces, and kidneys, showing no correlation with the concentration of bromadiolone. The pattern difference between bromadiolone and warfarin is primarily observed in their distribution across various tissues and organs. Bromadiolone exhibited a more consistent and organ-specific pattern of higher concentrations in the liver and kidneys, whereas warfarin showed a more varied and less predictable distribution across different tissues.

## 3. Discussion

By providing detailed insight into the distribution and toxic effects of warfarin and bromadiolone in birds, our work not only contributes to wildlife conservation, but also helps understand the broader environmental and public health implications of rodenticide use, as well as assisting forensic investigations. This is one of the first works dealing with this important problem, which is unfortunately becoming more and more common in Poland every year.

Poisoning by rodenticides in non-target animals is a serious environmental problem worldwide [2,8]. These agents are characterized by a prolonged duration of action, making weakened animals an easy target for predators, both mammals and birds, for which they can also be deadly. Moreover, the indirect exposure of raptors to rodenticides by eating animals lower down the food chain previously poisoned with rodenticides is suspected to be a significant contributor to bird mortality [2].

To date, research on secondary rodenticide poisoning in birds has focused mainly on large raptors [5,7,14,15]. However, also in this case, the routes of exposure and the frequency of such events are not fully known [2]. As for smaller birds, the issue of poisoning of rooks and carrion crows with rodenticides is poorly understood, and there are relatively little literature data on this subject. However, the rapid decline in the rook population in Poland, especially in rural areas, proves the importance of this problem [16].

Rooks and carrion crows are omnivorous birds, with a diet that includes cereal grains like oats, wheat, and barley, as well as small invertebrates such as snails and beetles. Importantly, from the perspective of rodenticide exposure, they also consume small rodents like mice and voles, both through hunting and scavenging [17,18,19].

Indirect rodenticide poisoning can occur in two ways. First, rodenticides have a prolonged effect on rodent bodies, which means that such rodents can continue to exist in the environment for some time after consuming the poison, and the weakening caused by the poison will make them easy prey for birds of prey [2]. The second possible route of exposure for the species we study is feeding on carrion—poisoned rodents that died at the surface may become easy prey for scavengers [20]. It is worth noting that both species studied by us are synanthropic birds that live in human environments both in cities and in villages near farms and arable fields [19]. The proximity of living close to people means that in addition to being exposed to indirect poisoning through the consumption of weakened or dead rodents as a result of rodent control campaigns, these birds can also be exposed to the direct consumption of poisoned baits put out to protect crops threatened by mice and rats [8,20]. In France, bromadiolone on farms was particularly widely used to control water vole infestations in agricultural fields [21]; research showed that the carrion crow was responsible for 66% of all the feeding on water vole carcasses [20]. Researchers indicate that this situation contributes to reducing the exposure of other birds of prey protected by EU Directive 2009/147/EC [22], but this is not a fully acceptable phenomenon [20]. It should be mentioned here that both rooks and carrion crows may be protected under the laws of individual countries; such a situation occurs, among others, in Poland, where rooks are under strict protection outside urban areas and partial species protection in urban areas, while the carrion crow is covered under strict species protection throughout Poland [23].

It should be noted that warfarin and bromadiolone are contained in products intended for professional users who have completed the appropriate training according to Act [24,25], but in reality this is not verified and such products can be purchased by anyone, even through popular online stores and auction sites. This raises the risk of using too high doses of rodenticides, which pose a threat to the environment, or using them contrary to their intended use, especially on small family farms, to intentionally poison animals considered pests, such as foxes or grain-eating birds. The problem of the easy availability of rodenticides intended for professional users has already been highlighted in the scientific literature in the context of poisonings of protected white-tailed eagles, but nothing has changed in this respect since then. The results presented are the first studies on rodenticide poisoning in rooks and carrion crows in Poland. They indicate the need for legal changes leading to limiting the availability of biocides intended for professional users.

As mentioned, most rodenticide exposure studies were conducted on large birds. In Poland, these studies included white-tailed eagles. For example, in Sell’s work [5], the poisoning of white-tailed eagles with bromadiolone, brodifacoum, difenacoum and flocoumafen was analyzed, showing the presence of bromadiolone and brodifacoum in almost all analyzed liver samples. Referring from these results to ours, it can be concluded that bromadiolone was present at a lower level than in the samples we tested because it ranged from 0.0025 µg/g to 0.903 µg/g (our results showed the presence of this compound from 1.229 ± 0.019 µg/ g to 16.659 ± 1.499 µg/g). On this basis, it can be assumed that rooks and carrion crows are more resistant to rodenticides or have a greater ability to accumulate. However, further research in this area is needed to reveal the physiological mechanism. Taking into account the threat posed by rodenticides to wild birds in general, it is indicated that the greatest threat comes from second-generation anticoagulants, which include bromadiolone. This problem occurs not only in Poland, but throughout the world. In Finland, 65% of the mammal and bird samples tested contained bromadiolone; it was the rodenticide that was detected the most frequently and also had the highest concentration [26]. In turn, in Germany, neighboring Poland, bromadiolone was the third-most frequently detected rodenticide in birds of prey, while surprisingly, warfarin, a first-generation anticoagulant, was not detected in any samples [27]. This shows that wild birds can be exposed to different rodenticides depending on local rodent control practices.

## 4. Materials and Methods

**Figure 5:**
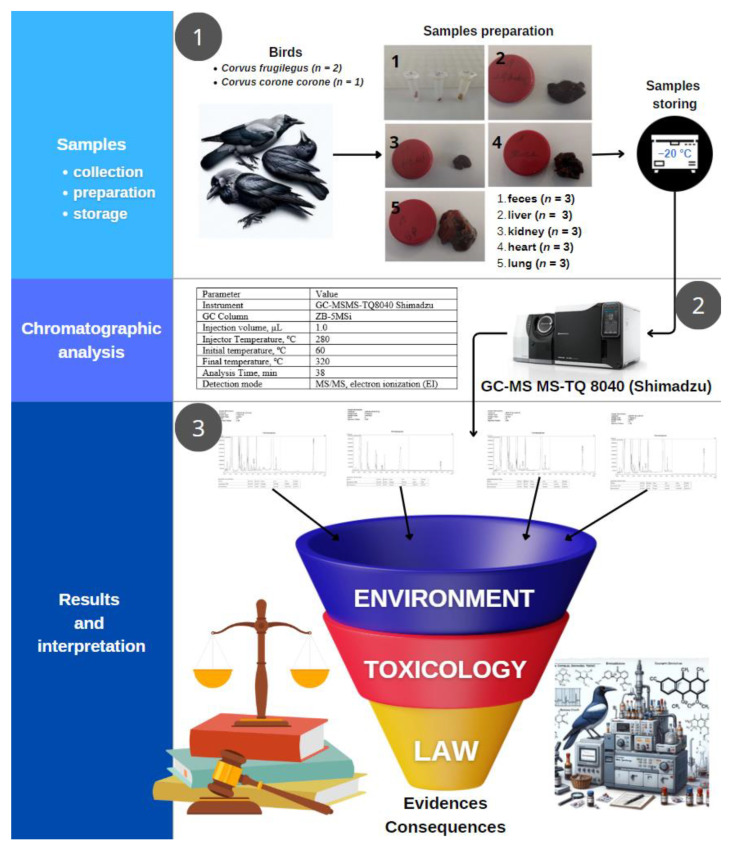
Workflow of the conducted studies.

The course of experiments is schematically presented in Figure 5.

### 4.1. Chemical and Reagents

The standards of warfarin (4-hydroxy-3-(3-oxo-1-phenylbutyl)chromen-2-one, CAS: 81-81-2) and bromadiolone (3-[3-[4-(4-bromophenyl)phenyl]-3-hydroxy-1-phenylpropyl]-4-hydroxychromen-2-one, CAS: 28772-56-7) were supplied by Sigma-Aldrich (Poznań, Poland). Ethyl acetate and methanol (both of LC/MS grade) used to prepare stock solutions were purchased from Merck (Darmstadt, Germany). Anhydrous sodium sulphate was bought from POCh (Gliwice, Poland). Pyridine, trimethylchlorosilane (TMCS) and a mixture of N,O-bis(trimethylsilyl)-trifluoroacetamide (BSTFA), with 1% TMCS used as the derivatization mixture, were obtained from Sigma-Aldrich (Poznań, Poland). Deionized water was purified using a Milli-Q system (Millipore Sigma, Bedford, MA, USA). Individual stock standard solutions and working solutions of standards, obtained through successive dilution of the stock solutions, were prepared in methanol. They were all kept under stable conditions at −20 °C (±2 °C).

### 4.2. Samples Collection and Storage

In the course of this research, no experimental procedures were conducted directly on live animals. The study only involved the examination of avian organs, which were provided by the Public Prosecutor’s Office. These organ specimens were obtained post-mortem, ensuring that our research adhered strictly to ethical guidelines concerning the use of animal subjects in scientific investigations. Our methodology was confined to the analysis of these donated samples, thus negating any requirement for direct animal testing or interaction. Samples of bird organs were collected under carefully controlled autopsy conditions performed by specialized personnel. This process was guided not only by strict compliance with quality standards, but also by the need to ensure the biological suitability and integrity of the samples. The target organs included the following: liver, kidney, right lung, heart, and faces. In order to capture a comprehensive biological profile that allows multi-aspect toxicological analysis, three different samples were collected for each organ type. To stop any biochemical changes and prevent degradation, samples were placed in a pre-cooled environment immediately after collection. The investigated forensic samples were evidence in the judicial investigation and were provided to us by the prosecutor’s office. Due to the importance of maintaining the integrity of the ongoing judicial case and preserving the confidentiality of evidence, we cannot disclose detailed information on the origin of the forensic samples investigated. These samples were provided to us by the prosecutor’s office and are being used as evidence in the investigation, hence the necessity of treating them with utmost confidentiality. Each sample was individually packaged in airtight containers labeled to prevent cross-contamination and moisture accumulation. Until analysis, samples were stored at –20 °C, a temperature strategically selected based on its effectiveness in preserving the cellular structure and molecular composition of biological tissues. Before analysis, samples were thoroughly thawed under controlled conditions to prevent rapid temperature changes that could affect sample properties. This gradual thawing process was necessary to maintain the physiological state of the samples and ensure that the analysis reflected their true biological state as closely as possible.

### 4.3. Sample Preparation

Immediately before analysis, the samples were thoroughly thawed at room temperature and manually homogenized with the addition of 1 mL of water. Accurately weighed portions were extracted three times with ethyl acetate. The combined extracts were centrifuged with the addition of anhydrous sodium sulfate. The supernatant was evaporated to dryness in a stream of nitrogen (XcelVap Evaporation/Concentration System, Horizon Technology, Salem, NH, USA) at room temperature and subjected to the derivatization procedure. To determine the optimal sample preparation conditions, the effect of solvent type (ethyl acetate or its mixture with n-propanol (85/15 *v*/*v*)), number of extraction cycles, and amounts of sodium sulphate and tissue samples on the recovery of analytes were examined. To obtain the trimethylsilyl (TMS) derivative of warfarin (bromadiolone does not derivatize), a slightly modified procedure described by Paprotny et al. [28] was used. Briefly, the dry residue was dissolved in 100 µL of pyridine, and then 25 µL of a derivatization mixture consisting of BSTFA with 1% TMCS catalyst was added. The tightly closed vial was mixed thoroughly using a vortex (10 s) and then heated at 70 °C for 60 min. After this time, after adding 625 µL of *n*-hexane, the contents of the vial were thoroughly mixed using a vortex (30 s), transferred to an Eppendorf tube and centrifuged (11,000× *g*, 5 min). Finally, the supernatant was transferred to a glass autosampler vial and subjected to chromatographic analysis.

### 4.4. GC-MS/MS Analysis and Its Optimization

The Shimadzu GC-MS system (Kyoto, Japan) was used. It is composed of an AOC-6000 autosampler (Shimadzu) and a gas chromatograph with a tandem mass spectrometer detector (GCMS-TQ8040). The samples (1 µL, splitless) injected using an AOC-6000 autosampler were separated on a Zebron ZB-5MSi fused-silica capillary column (30 m × 0.25 mm i.d., 0.25 µm film thickness; Phenomenex). Helium (grade 5.0) was used as the carrier gas and argon (grade 5.0) was used as the collision gas. The column flow was 1.08 mL/min. The injector was set to the high-pressure mode (200.0 kPa for 1.1 min; the column flow at an initial temperature of 280 °C was 3.50 mL/min). The initial column temperature of 60 °C was held for 5 min, and then raised to 320 °C. The final temperature was maintained for 5 min. The total analysis time was 38 min. The ion source and interface temperatures were 200 °C and 280 °C, respectively. The mass spectrometer was operated in the multiple reaction monitoring (MRM) mode using the electron ionization (EI) at 70 eV. To determine the optimal GC-MS/MS conditions, the effects of the injector temperature (200 or 250 or 280 °C) and the temperature increase per time unit (7.5 °C per 1 min or 15 °C per 1 min) were investigated. To establish the MS/MS operating conditions, the standard solutions of each analyte were determined separately. For each compound, mass transitions of the most sensitive or selective precursor ions were optimized regarding their product ions and corresponding collision energy.

### 4.5. Method Validation and Statistical Analysis

Validation experiments were carried out on chicken offal samples purchased from a local butcher shop. Chicken feces samples were collected at a friendly farm. All samples were previously checked for the absence of the determined compounds. The method was validated in terms of linearity, the limit of detection (LOD), the limit of quantification (LOQ) and the intra- and inter-day precision and accuracy measurements [29,30]. To evaluate the method linearity, three replicated analytical procedures were performed for five examined concentration levels. The peak areas were used for the quantification of the calibration curves. In order to estimate the LOD and the LOQ, the extracts spiked with the analytical reference standards were injected. The LOD and LOQ were considered to be signal-to-noise ratios equal to 3 and 10, respectively. The intra- and inter-day precisions and accuracies were evaluated using statistical analysis of the quantitative results (obtained on the same day and on three different days) for five independent samples containing test compounds (10 ng/mL). Recovery levels were investigated using blank samples spiked with the reference standards of the test compounds at three different concentration levels (1, 25 and 75 ng/mL). They were calculated as the percentage of the analyte response after sample work-up compared to that of a solution containing the analyte at a concentration corresponding to 100% recovery. In order to determine whether there was a significant difference between the recovery percentages at individual analyte concentration levels, a one-way analysis of variance (ANOVA) was performed. The linearity of the assay was calculated by the least squares method and expressed as the coefficient of determination (*R*^2^). Calibration plots were prepared using the blank samples spiked with analytes of 0.1, 0.5, 1, 25, 50 and 100 ng/mL.

## 5. Conclusions

This study has provided crucial insights into the unintended environmental and impacts of anticoagulant rodenticides, warfarin and bromadiolone. By analyzing tissue samples from rooks and carrion crows, we identified significant variations in the concentration of these toxic compounds, with bromadiolone particularly accumulating in the liver and kidneys. Despite regulatory measures that restrict these substances to professional use, their widespread availability presents a substantial risk to wildlife and public health due to unintentional poisoning. Future research should focus on comparative studies across different wildlife species, longitudinal studies on the long-term ecological impacts, and assessments of regulatory effectiveness. Addressing these areas can help mitigate the risks posed by rodenticides to non-target wildlife and contribute to broader conservation and public health efforts.

## Figures and Tables

**Table 1:** Results of validation for the presented method.

Tested Parameter		Warfarin	Bromadiolone
Linearity		0.9999	0.9989
Precision	Intra-day	5.73	6.78
(%RSD)	Inter-day	7.82	5.99
Accuracy	Intra-day	96.5	94.8
(%RSD)	Inter-day	98.6	102.0
Recovery (%)		98.7	95.6
LOD (ng/mL)		0.1	0.2
LOQ (ng/mL)		0.3	0.6

## Data Availability

The data that support the findings of this study are not openly available due to reasons of sensitivity and are available from the corresponding author upon reasonable request.
